# Effects of tamoxifen inducible MerCreMer on gene expression in cardiac myocytes in mice

**DOI:** 10.20517/jca.2021.30

**Published:** 2022-01-05

**Authors:** Leila Rouhi, Siyang Fan, Sirisha M. Cheedipudi, Melis Olcum, Hyun-Hwan Jeong, Zhongming Zhao, Priyatansh Gurha, Ali J. Marian

**Affiliations:** 1 Center for Cardiovascular Genetics, Institute of Molecular Medicine and Department of Medicine, University of Texas Health Sciences Center at Houston, Houston, TX 77030, USA; 2 Heart Center & Beijing Key Laboratory of Hypertension, Beijing Chaoyang Hospital, Capital Medical University, Beijing 100020, China; 3 Center for Precision Health, School of Biomedical Informatics and School of Public Health, The University of Texas Health Science Center at Houston, Houston, TX 77030, USA

**Keywords:** Transcriptome, gene expression, tamoxifen, Cre recombinase, MerCreMer, interferon, inflammation, TP53

## Abstract

The Cre-LoxP technology, including the tamoxifen (TAM) inducible MerCreMer (MCM), is increasingly used to delineate gene function, understand the disease mechanisms, and test therapeutic interventions. We set to determine the effects of TAM-MCM on cardiac myocyte transcriptome.

Expression of the MCM was induced specifically in cardiac myocytes upon injection of TAM to myosin heavy chain 6-MCM (*Myh6-Mcm*) mice for 5 consecutive days. Cardiac function, myocardial histology, and gene expression (RNA-sequencing) were analyzed 2 weeks after TAM injection. A total of 346 protein coding genes (168 up- and 178 down-regulated) were differentially expressed. Transcript levels of 85 genes, analyzed by a reverse transcription-polymerase chain reaction in independent samples, correlated with changes in the RNA-sequencing data. The differentially expressed genes were modestly enriched for genes involved in the interferon response and the tumor protein 53 (TP53) pathways. The changes in gene expression were relatively small and mostly transient and had no discernible effects on cardiac function, myocardial fibrosis, and apoptosis or induction of double-stranded DNA breaks.

Thus, TAM-inducible activation of MCM alters cardiac myocytes gene expression, provoking modest and transient interferon and DNA damage responses without exerting other discernible phenotypic effects. Thus, the effects of TAM-MCM on gene expression should be considered in discerning the bona fide changes that result from the targeting of the gene of interest.

## Graphical Abstract

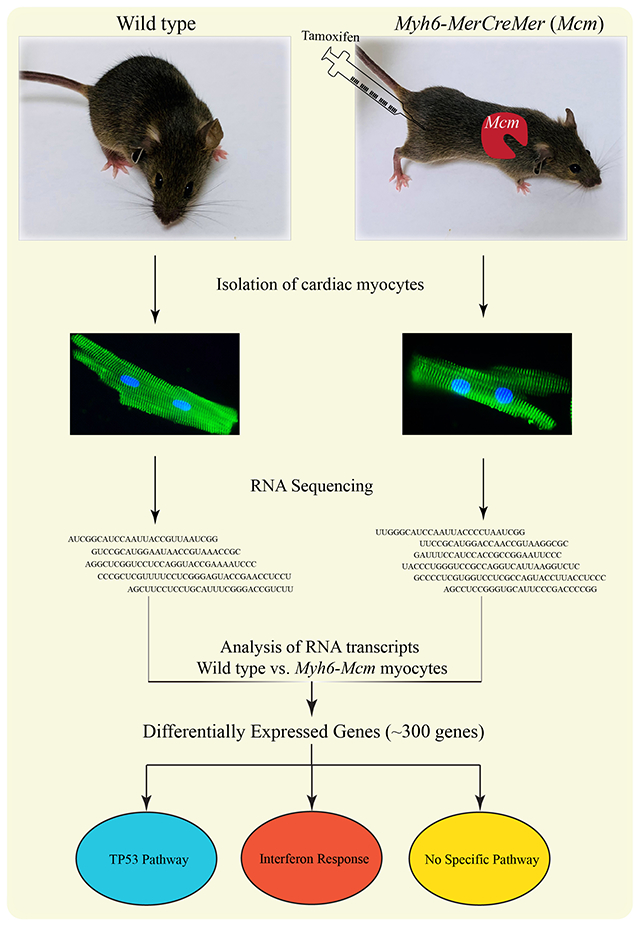

The seminal discovery of the bacteriophage Cre recombinase-Lox system has made it possible to delete, insert, or rearrange the specific genomic loci flanked by the *LoxP* sequences^[1]^. Expression of the Cre recombinase under the transcriptional regulation of a cell type-specific promoter enables knocking out the gene of interest, flanked by the *LoxP* sites, also called the floxed gene or allele, in the desired cell type in a given organ. The system has been instrumental in delineating the biological functions of a very large number of genes and generating organismal models of human diseases, including cardiovascular aging.

A commonly used Cre-LoxP system in cardiovascular sciences exploits the myosin heavy chain 6 (*Myh6*) promoter/enhancer to express the Cre recombinase and induce efficient recombination of the floxed gene, specifically in cardiac myocytes^[2]^. Persistent expression of the bacteriophage protein Cre in cardiac myocytes, however, imparts untoward effects, including cytotoxicity and cardiac dysfunction^[3]^. To circumvent the toxicity, a ligand inducible Cre-LoxP system has been developed wherein an inactive fusion protein, comprised of the Cre recombinase flanked by the ligand binding domain of the estrogen receptor, referred to as MerCreMer (MCM), is expressed^[4,5]^. Administration of tamoxifen (TAM) or 4-hydroxytamoxifen activates the MCM protein, leading to its nuclear translocation and induction of recombination of the floxed gene^[4,5]^. Expression of MCM under the *Myh6* promoter/enhancer and administration of TAM induces efficient recombination specifically in cardiac myocytes in mice^[6]^. TAM, however, when used at high doses, induces a transient dose-dependent cardiac dysfunction^[7,8]^. Consequently, a lower dose of TAM is used to induce efficient recombination and generate models of human diseases^[9]^. However, there is a concern that administration of TAM and expression of the MCM protein could affect gene expression in cardiac myocyte and confound interpretation of the findings, hindering from identifying the bona fide changes that occur upon knock out of the gene of interest.

To determine changes in cardiac myocyte gene expression upon TAM-induced activation of the MCM fusion protein, the myosin heavy chain 6-MCM (*Myh6-Mcm*) mice [B6.FVB(129)-A1cf^Tg(Myh6-cre/Esr1*)1Jmk^/J] were treated with subcutaneous injection of TAM at 30 mg/kg/day for 5 consecutive days starting at the post-natal day 14. The time point was chosen as cardiac myocytes progressively lose their proliferative capacity after birth and are almost exclusively non-proliferative during adult life^[10]^. The dose was selected to reduce the risk of transient toxicity associated with higher doses of TAM administration^[7,8]^. All experiments were performed in accord with the NIH Guide for the Care and Use of Laboratory Animals, and the protocols were approved by the Institutional Care and Use Committee (Protocol # AWC-18-0048).

Genotyping was performed by PCR of mouse tail genomic DNA. The list of oligonucleotide primers used for the genotyping is provided in Supplementary Table 1. Expression of the *Mcm* transgene was detected by reverse transcription-polymerase chain reaction (RT-PCR) using primers that target exon 6-7 of the estrogen receptor (*Esr1*) gene that is part of the MCM construct. Transcript levels of the *Esr1* gene were significantly increased in the *Myh6-Mcm* myocyte RNA compared to the wild type (WT) [Figure 1A]. There were no differences in the survival rates between the WT and *Myh6-Mcm* mice injected with TAM up to 9 months of age. Likewise, there were no differences in gross cardiac morphology and the heart weight/body weight ratio between the two groups [Figure 1B]. Cardiac function was assessed prior to the analysis of gene expression at 4 weeks of age by echocardiography, as published^[11,12]^. There were no differences in the echocardiographic indices of cardiac size and function at 4 weeks of age between the WT and TAM injected *Myh6-Mcm* mice [Table 1]. Mice with normal cardiac size and function were used to isolate cardiac myocytes and extract RNA for gene expression analysis. The time point was chosen to reduce potential confounding effects of cardiac dysfunction on gene expression and identify changes that are due to TAM injection and MCM expression.

Cardiac myocyte transcripts were analyzed by RNA-sequencing (RNA-Seq), as published^[11–13]^. In brief, total RNA was extracted from mouse ventricular cardiac myocytes, and samples with an RNA Integrity Number of > 8 were depleted from ribosomal RNA and used to generate strand-specific sequencing libraries. The libraries were sequenced as 75 bp paired-end reads on an Illumina HiSeq 4000 instrument.

FastQC was used to check the quality of RNA sequence reads, and Spliced Transcripts Alignment to a Reference was used to process the sequence alignment with the mouse reference genome build mm10 and to generate gene read counts^[14,15]^. The uniquely aligned read pairs were annotated for gene features using *GENCODE* gene model (https://www.gencodegenes.org/mouse/). The average number of total reads (54.7 ± 4.4 million reads), uniquely mapped reads (41.2 ± 3.8 million reads), and percent uniquely mapped reads (75.3% ± 2.5%) indicated a high quality of RNA-sequencing and did not differ between the two genotypes [Supplementary Table 2]. Genes with 1 read count per million (CPM) in at least 5 samples were analyzed to identify the differentially expressed genes (DEGs) using the Limma-Voom (variance modeling at the observation-level) with sample weight analysis program in the R package^[16,17]^. The analysis was repeated using DeSeq2 in order to reduce the platform-dependent findings. Benjamini-Hochberg false discovery rate (FDR)-adjusted *P* value of < 0.05 were considered significant.

The transcript levels were assessed by principal component analysis to assess genotype-dependent clustering, which showed a distinct separation of the *Myh6-Mcm* and WT myocyte transcripts [Figure 1C]. Transcript levels of 346 protein coding genes were differentially expressed, which were comprised of 168 upregulated and 178 downregulated genes, whereas transcript levels of 10,704 genes were unchanged [Figure 1D]. A heat map of the DEGs, generated using the normalized CPM values, is depicted in Figure 1E. *Gbp2b*, *Lipo2*, and *Trnp1*, encoding guanylate binding protein 2, lipase member O2, and TMF1 regulated nuclear protein 1 were among the most upregulated, whereas *Slc28a2b*, *Strit1*, and *Eif3j2*, encoding sodium/nucleoside cotransporter, small transmembrane regulator of ion transport 1, and eukaryotic translation initiation factor 3, subunit J2 were among the most downregulated genes [Supplementary Table 3].

Analysis of the RNA-Seq data using DeSeq2 led to the identification of 358 DEGs (excluding *Esr1*, which originates from the MerCreMer construct), comprised of 156 upregulated and 202 downregulated genes (q < 0.05). As in the analysis by Limma-Voom software, the top DEGs were *Gbp2b*, *Lipo2*, and *Trnp1*, whereas the *Strit1*, *Angptl4* (angiopoietin-like 4), and *Mme* (membrane metalloendopeptidase) comprised the top downregulated genes. The list of the DEGs, identified using DeSeq2, is also included in Supplementary Table 3.

To test for confirmation of the RNA-Seq data, transcript levels of 85 genes were analyzed by RT-PCR in independent samples. Changes in transcript levels of the selected genes, presented as fold change, were mostly concordant between the RNA-Seq and RT-PCR datasets and showed a significant correlation [Figure 1F]. Accordingly, with the exception of a few genes, those that were upregulated in the RNA-Seq dataset were also upregulated in the RT-PCR datasets and conversely, those that were downregulated in the RNA-Seq were also downregulated in the RT-PCR datasets [Figure 1F]. However, transcript levels of several genes showed discordance between the two techniques, which were depicted in Figure 1F. The discordance likely reflects the differences in quantifying the transcript levels of each gene between the two techniques. Whereas the RT-PCR amplifies a specific exon or exons of a gene, which are covered by the oligonucleotide primers, the RNA-Seq technique considers the total mapped short sequence reads of each gene, which are often non-homogeneous across the exons and the approach is ignorant of the transcript isoforms. Consequently, RT-PCR reflects transcript levels of a specific exon(s), which might be over- or under-represented in the mapped reads in the RNA-Seq data. A heat map of the transcript levels of genes analyzed by the two methods is depicted in Figure 1G.

The DEGs were analyzed to predict the dysregulated transcriptional regulators (TRs) using the upstream regulator analysis function of the Ingenuity Pathway Analysis software (IPA®, QIAGEN Redwood City). TRs are showing a *P* value of < 0.05 for overlap with the IPA target genes, and a predicted Z score of < −2 or > 2 was considered dysregulated. It is important to note that the number of the DEGs that overlapped with genes in each upstream regulator pathway was relatively small [Figure 2A]. Notably, 16 and 11 upregulated genes that overlapped with the genes known to be regulated by the interferon-gamma (INFγ) and platelet-derived growth factor bb, respectively, suggesting activation of these regulators. Likewise, 10 genes whose transcript levels were suppressed in the TAM/*Myh6-Mcm* myocytes were known to be regulated by mitogen-activated protein kinase 1, suggesting suppression of this kinase pathway. Likewise, overrepresentation analysis of the DEGs showed enrichment of the tumor-suppressor gene tumor protein 53 (TP53) (*n* = 10 genes), interferon α and γ (7 and 8 genes, respectively) pathways, suggesting activation of the inflammatory/immune responses [Figure 2B]. In contrast, the DEGs predicted suppression of the mechanistic target of rapamycin complex 1 (mTORC_1_) signaling pathway [Figure 2C]. Contributions of the DEGs to the predicted dysregulated biological pathways are depicted in the Circos map in Figure 2D. Furthermore, gene set enrichment analysis was concordant with the pathway analysis, predicting activation of the interferon response to injection of TAM and activation of MCM fusion protein in cardiac myocytes [Figure 2E].

The analysis of the DEGs using a stringent criterion of 1% FDR showed differential expression of only 78 genes (by Limma-Voom), comprised of 37 upregulated and 41 downregulated genes [Supplementary Table 3]. Likewise, analysis of gene expression using DeSEq2 with FDR cut off of < 0.01 identified only 177 DEGs [Supplementary Table 3]. Overall, the number of DEGs with an FDR of < 0.01 that overlapped with the target genes of the TRs was too small to make firm conclusions.

To determine phenotypic consequences of altered gene expression upon administration of TAM and expression of MCM, myocardial fibrosis, apoptosis, and double-stranded DNA breaks (DSB) were analyzed by histological techniques and immunoblotting. Likewise, cardiac size and function were analyzed by echocardiography, which were normal as described earlier [Table 1]. In brief, thin myocardial sections were stained with picrosirius red, and collagen volume fraction (CVF) was calculated as the percentage of the areas stained for the picrosirius red, as published^[18,19]^. There was no difference in the myocardial CVF between the two groups at 4 weeks of age [Figure 3A and B]. Likewise, myocardial apoptosis was analyzed by the terminal deoxynucleotidyl transferase deoxyuridine triphosphate nick end labeling (TUNEL) assay using *in-situ* cell death detection Fluorescein kit (Roche Cat#11684795910), as published^[11,19]^. The percentage of nuclei stained positive for the TUNEL, determined in approximately 20,000 cells per mouse, was not different between the two groups [Figure 3C and D]. Finally, the expression of phospho-histone 2A family member X (pH2AFX), a marker for DSBs, was analyzed by immunofluorescence and immunoblotting. There were a scant number of myocardial cells that were stained for the expression of phospho-H2AFX in each group [Figure 3E and F]. The expression levels of this protein were not different between the wild type and *Myh6-Mcm* mice, injected with TAM [Figure 3G and H].

To determine whether dysregulated gene expression persisted in cardiac myocytes, transcript levels of a dozen genes, which were differentially expressed at 4 weeks, including the *Mcm* transgene, were re-examined 6 months after treatment of the *Myh6-Mcm* mice with TAM. There were no differences in the transcript levels of the selected genes at 6 months of age, except that the *Mcm* transgene, which was represented by the transcript of *Esr1*, and transcript levels of *Abhd1* and *Armcx4*, encoding abhydrolase domain containing 1 and armadillo repeat-containing X-linked 4, respectively, were modestly reduced [Figure 4A]. Likewise, cardiac function assessed at 6 months of age was normal in the TAM-treated *Myh6-Mcm* mice [Table 2]. Histological examination of the myocardium also did not show evidence of increased myocardial fibrosis and apoptosis at 6 months of age [Figure 4B–E]. Thus, the findings indicate the transient nature of dysregulated gene expression in cardiac myocytes upon administration of TAM and induction and activation of MCM in cardiac myocytes.

The design of the study, including the selection of the time point and the echocardiographic assessment of cardiac function in mice in the RNA-Sequencing experiments, reduces the potential confounding effects of transient cardiac dysfunction, which is known to occur after administration of TAM or 4 hydroxy tamoxifen in the *Myh6-Mcm* mice^[7,8]^. Likewise, analysis of gene expression in the absence of discernible histological changes also reduced the potential confounding effects of myocardial fibrosis and apoptosis on the transcript levels. Moreover, the sequencing depth was high (~55 M reads per sample), and the DEGs were analyzed using two different bioinformatic algorithms with a significance FDR set at 0.05 and 0.01, both identifying a modest enrichment of the DEGs in the immunity and inflammatory pathways. Moreover, the RNA-sequencing findings for selected DEGs were mostly corroborated in independent samples by an alternative method.

The data provide evidence for a modest activation of the interferon response, without a clear distinction between type I or II responses, as the DEGs were members of both responses. Nevertheless, activation of the interferon pathway is in accord with the innate immune system against external pathogens, such as the bacteriophage Cre recombinase and/or induction of DNA recombination independent of genuine *LoxP* sites^[20]^. The TP53 pathway was also predicted to be activated, based on the DEGs, likely in response to the genomic DNA damage caused by the Cre recombinase. The latter is reflected by the increased transcript levels of the DNA damage response genes *Xpc*, *Ier5*, *Ddit3*, and *Abhd4* in cardiac myocytes isolated from the TAM treated *Myh6-Mcm* mice. Activation of the DNA damage response genes, although modest, suggests that the Cre-mediated recombination event also occurs even in the absence of *LoxP* sites and likely because of the presence of pseudo-LoxP sites in the mammalian genome^[20–22]^. However, the magnitude of the DNA damage seems to be modest and limited to changes in the transcript levels of a few genes, and hence, it was not sufficient to induce a phenotype or to be detected by staining of the myocardial section for DSB marker pH2AFX. The DEGs also predicted suppression of the mTORC*1* pathway, albeit the number of the DEGs that overlapped with genes in the mTORC1 pathway was relatively small, precluding from making firm conclusions.

The study has a number of weaknesses. First and foremost, it is important to note that the predicted dysregulated biological pathways or transcriptional regulators were based on a relatively small number of DEGs, and therefore, might not be sufficiently robust, despite the conventionally significant q values or Z scores. The RNA-Seq experiments were performed at one time point and RT-PCR at two time points of 4 weeks, coinciding with RNA-Seq time point, and at 6 months, the latter to assess persistence of the changes. Transcriptomic changes were mostly resolved at the latter time point, and there was no discernible phenotype despite persistent expression of the transgene (inactive MCM protein). Thus, the exact duration of the transcriptomic changes in cardiac myocytes remains undetermined but is less than 6 months as would be expected given the expected turnover rates of mRNA and MCM protein after discontinuation of TAM. It also merits noting that despite changes in the gene expression observed at 4 weeks, including persistent expression of the *Mcm* transgene, there was no evidence of cardiac dysfunction, myocardial fibrosis, apoptosis, or DSBs in the *Myh6-Mcm* mice. The latter set of findings indicates that transient activation of the Cre protein in cardiac myocytes and persistent expression of the inactive MCM fusion protein under the transcriptional regulation of the *Myh6* promoter are largely inconsequential on cardiac function and myocardial histology. Furthermore, the absence of functional and histological phenotype, despite changes in gene expression, suggests that coordinated dysregulation of a larger number of genes in each biological pathway might be necessary to induce the phenotype. Given the relatively high depth of the RNA-Seq reads and the absence of a discernible histological and functional phenotype, some of the changes in the transcript levels might be transcriptional and technical noise. Whereas there were no sex-specific differences in gene expression in cardiac myocytes between the WT and *Myh6-Mcm* mice, the study was not designed to address the effects of genotype-by-sex interactions on gene expression. Moreover, the findings of the study are restricted to the relatively low dose of TAM used in these studies, the mouse strain, and the Myh6 promoter/enhancer. Finally, the findings are specific to myocyte gene expression and might not be relevant to the effects of TAM/MCM on gene expression in other cell types.

In conclusion, the findings, for the first time to our knowledge, define the transcriptomic signature of the TAM injection and expression of the MCM fusion protein in cardiac myocytes, which were validated for a number of selected genes in independent experiments by an alternative method. The findings emphasize the necessity of including the *Myh6-Mcm* mice treated with TAM as a control group in experiments using the MCM system for genetic manipulation in mice. The dataset is expected to enable the investigators to discern the bona fide changes that result from the intended genetic recombination from those that are fortuitously induced by the TAM/MCM system, and therefore, reducing the risk of spurious conclusions.

## Supplementary Material

Supplementary Materials

## Figures and Tables

**Figure 1.**
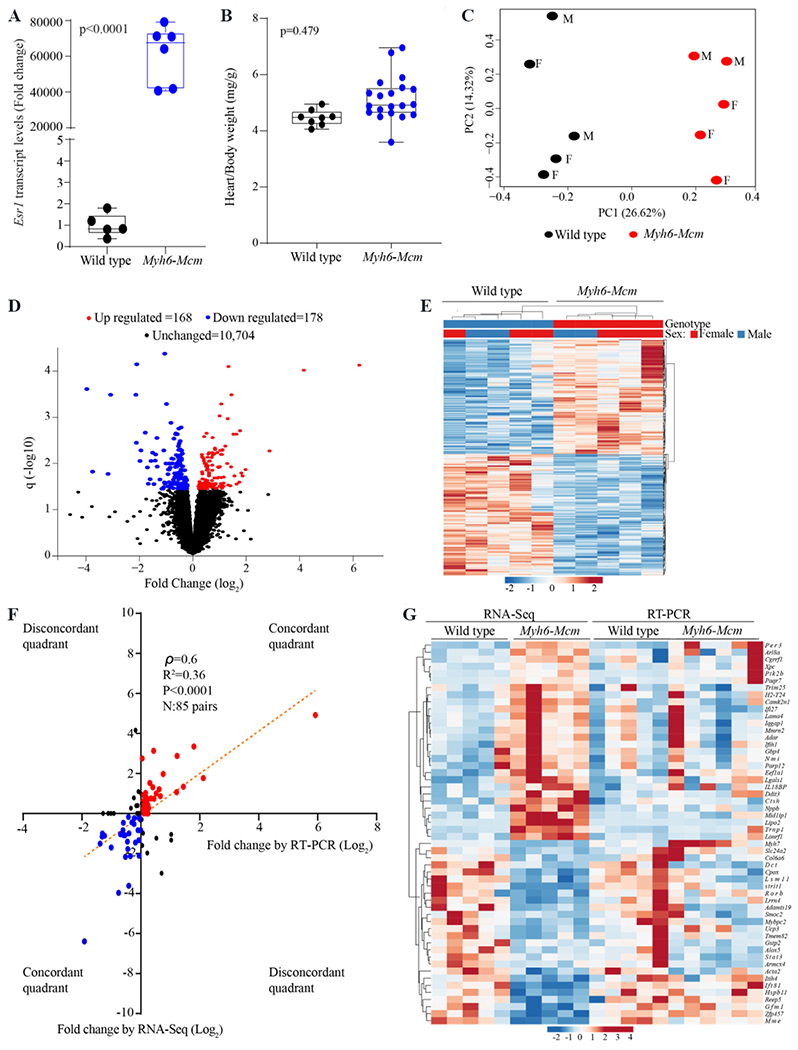
Effects of tamoxifen (TAM) injection and expression of the MerCreMer (MCM) transgene protein on cardiac myocyte transcripts. (A) Levels of the transgene (*Mcm*) transcripts detected by RT-PCR of the ligand binding domain of the estrogen receptor (*Esr1*), showing markedly increased levels in the TAM injected *Myh6-Mcm* relative to wild type (WT) mouse myocytes. (B) Heart/body weight ratio showing no difference between the two groups. (C) Principal component analysis (PCA) of the cardiac myocyte transcripts showing distinct separation of the transcripts of myocytes isolated from the WT and *Myh6-Mcm* mice. (D) Volcano plot of transcripts identifying the differentially expressed genes (DEGs). The up-regulated genes are shown in red, the downregulated ones in blue, and those unchanged in black. (E) Heat map of the DEGs, showing distinct genotype-dependent categorization. (F) Pearson correlation plot showing a significant correlation in the changes in the transcript levels of 85 genes between the WT and *Myh6-Mcm* myocytes, as detected by RNA-sequencing (RNA-Seq) and reverse transcription-polymerase chain reaction (RT-PCR) methods in independent samples. Changes between the genotypes are presented as fold change (Log2). (G) Heat map of the transcript levels of selected genes in the WT and *Myh6-Mcm* myocytes as quantified by the RNA-Seq and RT-PCR in independent samples.

**Figure 2.**
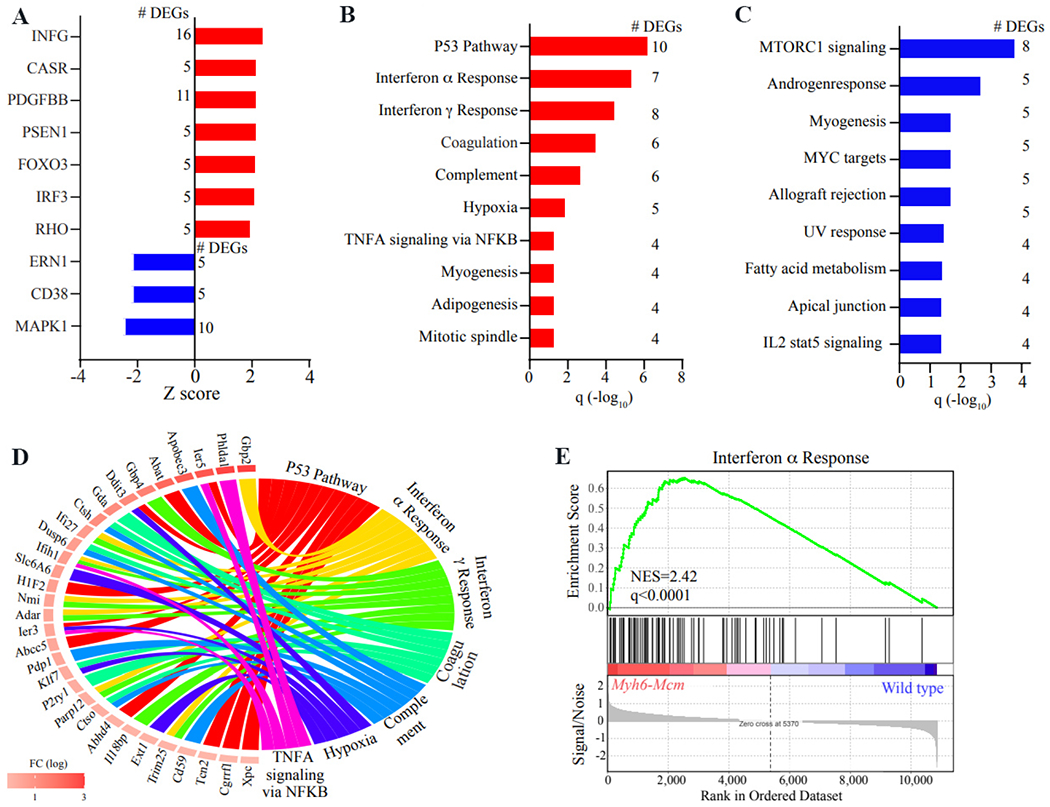
Predicted changes in the regulators of gene expression and biological pathways. (A) Panel A illustrated the transcriptional regulators (TRs), which are predicted to be activated or suppressed based on the number of differentially expressed genes (DEGs). The latter is depicted next to each TR. Red indicated predicted activation and blue predicted suppression. A Z score of < −2 and greater > 2 is considered significant. (B) The list of the biological pathways obtained from the overrepresentation (OR) analysis of the upregulated genes is depicted in the graph, along with the number of DEGs that overlaps with the genes in that pathway. (C) The list of biological pathways was obtained from the OR analysis of the downregulated genes in the *Myh6-Mcm* myocytes. The number of the DEGs that overlap with the genes in each pathway is listed next to each pathway. (D) Circos plot depicting the predicted activated biological pathways and contribution of the genes to the dysregulated pathways. (E) Gene set enrichment analysis (GSEA) showing enrichment of the genes in the interferon alpha pathway in the *Myh6-Mcm* myocytes.

**Figure 3.**
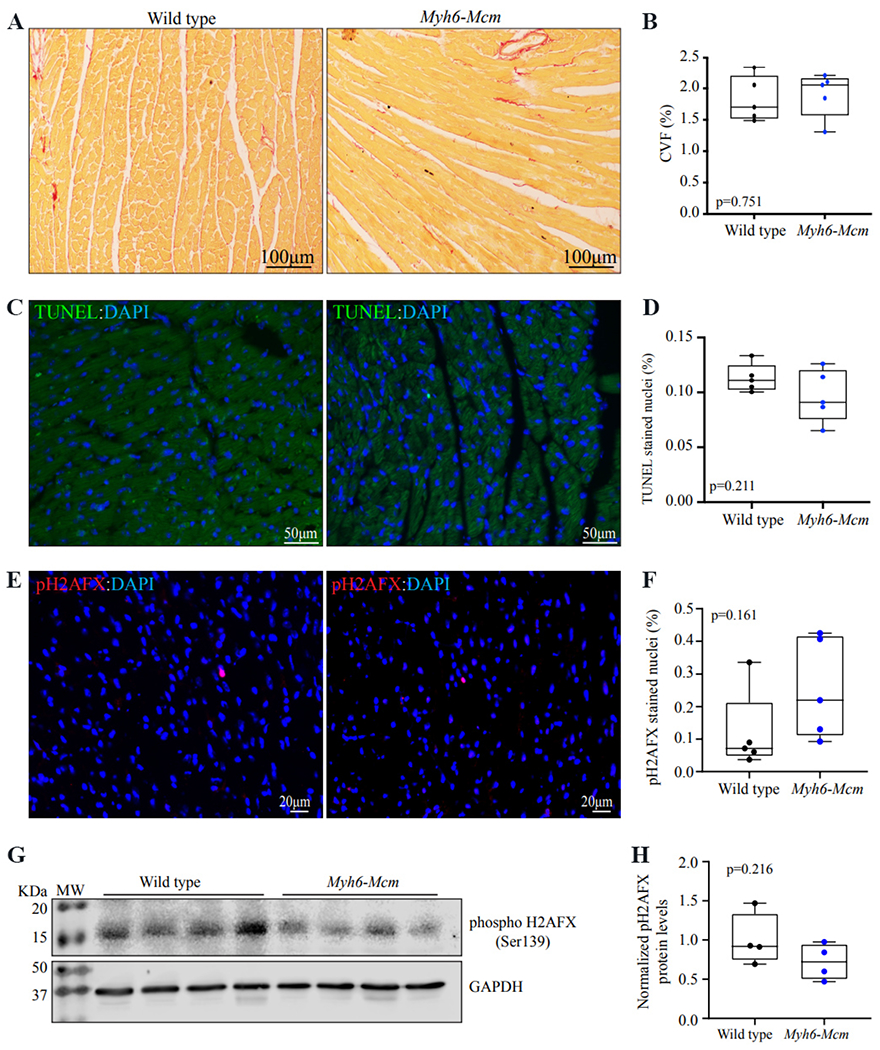
Histological evaluation of the myocardium in the *Myh6-Mcm* mice injected with tamoxifen (TAM) at 4 weeks of age. (A) Picrosirius red-stained thin myocardial sections in the wild type and *Myh6-Mcm* (injected with TAM) mice at 4 weeks of age, showing no evidence of myocardial fibrosis. (B) Quantitative data of myocardial fibrosis presented as collagen volume fraction (CVF) in the experimental groups. (C) Assessment of apoptosis by the transferase deoxyuridine triphosphate (dUTP) nick end labeling (TUNEL) assay in the myocardial sections from the wild type and *Myh6-Mcm* (injected with TAM) mice, showing rare cells stained for TUNEL in green. Nuclei are stained with 4′,6-diamidino-2-phenylindole (DAPI) and shown in blue color. (D) Quantitative data showing the percentage of nuclei stained for TUNEL in each experimental group. (E) Immunofluorescence panel showing thin myocardial sections stained for phospho-the histone protein family member X (H2AFX) in the wild type and *Myh6-Mcm* (injected with TAM) mice, showing scattered positive cells. (F) Quantitative data showing the percent of phospho-H2AFX stained nuclei in the experimental groups. (G) Immunoblot analysis of cardiac myocyte protein extracts of wild type and *Myh6-Mcm* (injected with TAM) mice detecting phospho-H2AFX and glyceraldehyde-3-phosphate dehydrogenase (GAPDH) [Supplementary Figure 1]. (H) Quantitative analysis of immunoblots detecting the phospho-H2AFX protein levels normalized to the GAPDH protein levels.

**Figure 4.**
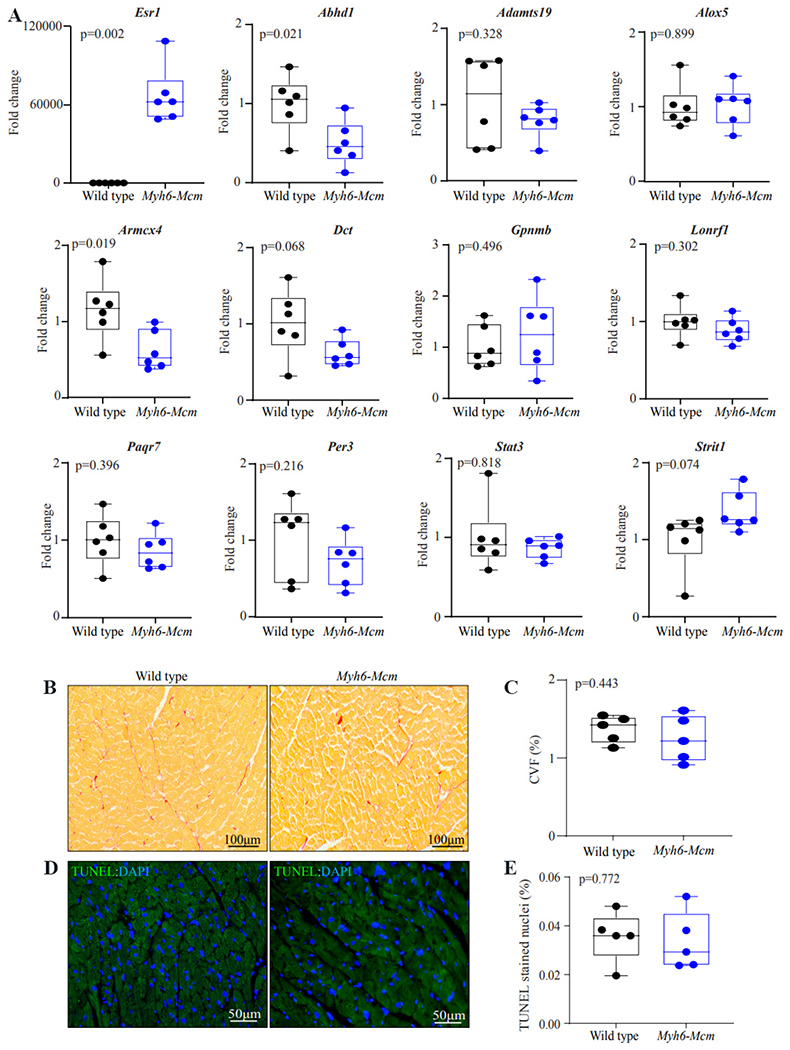
Gene expression and myocardial histology in 6 months old wild type and *Myh6-Mcm* mice. (A) Transcript levels of a dozen genes analyzed by reverse transcription-polymerase chain reaction 6 months after induction of activation of the MerCreMer protein upon injection of tamoxifen for 5 consecutive days at 2 weeks of age. Transcript levels of *Esr1*, representing the transgene, remained significantly elevated at 6 months of age. Transcript levels of *Abhd1* and *Armcx4* were also modestly reduced. (B) Picrosirius red-stained thin myocardial sections in the 6 months old wild type and *Myh6-Mcm* (injected with tamoxifen) mice, showing no evidence of myocardial fibrosis. (C) Quantitative data of myocardial fibrosis presented as CVF. (D) Myocardial panels from 6 months old mice stained for the TUNEL assay. Rare nuclei stained for the TUNEL (green) were detected. Nuclei are shown in blue upon DAPI staining. (E) Quantitative data showing the percentage of nuclei stained for TUNEL. CVF: Collagen volume fraction; TUNEL: transferase deoxyuridine triphosphate (dUTP) nick end labeling.

**Table 1.** Echocardiographic indices of cardiac size and function at 4 weeks of age

	4 weeks		
	WT	*Myh6-Mcm*	*P* (*t*-test)
*n*	9	10	NA
M/F	4/5	5/5	0.808*
Age (days)	28.22 ± 0.44	28.7 ± 0.67	0.089
Body weight (g)	16.24 ± 1.82	15.64 ± 1.26	0.407
HR (bpm)	660.04 ± 29.14	674.18 ± 38.22	0.381
AWT (mm)	0.36 ± 0.02	0.34 ± 0.02	0.038^#^
LVPWT (mm)	0.36 ± 0.02	0.36 ± 0.34	0.172
LVEDD (mm)	2.81 ± 0.3	2.96 ± 0.19	0.198
LVEDDI (mm/g)	0.112 ± 0.14	0.112 ± 0.20	0.114
LVESD (mm)	1.52 ± 0.15	1.54 ± 1.6	0.227
LVFS (%)	45.73 ± 2.29	46.03 ± 2.45	0.782
LV Mass (mg)	18.27 ± 4.68	18.55 ± 1.95	0.865
LVMI (mg/g)	1.12 ± 0.24	1.19 ± 0.12	0.461

* Denotes *P* value obtained by chi square test.

# Denotes *P* < 0.05.

*Myh6-Mcm*: Myosin heavy chain 6-MerCreMer; F/M: female/male; HR: heart rate; bpm: beats per minute; AWT: anterior wall thickness; LVPWT: left ventricular posterior wall thickness; LVEDD: left ventricular end diastolic diameter; LVEDDi: LVEDD indexed to body weight; LVESD: left ventricular end systolic diameter; LVFS: left ventricular fractional shortening; LVM: left ventricular mass; LVMI: LVM indexed to body weight.

**Table 2.** Echocardiographic indices of cardiac size and function at 6 months of age

	6 months		
	WT	*Myh6-Mcm*	*P* (*t*-test)
*n*	16	11	
M/F	9/7	6/5	0.930*
Age (days)	180.38 ± 0.50	180.64 ± 1.21	0.442
Body weight (g)	30.66 ± 4.05	30.43 ± 5.51	0.899
HR (bpm)	606.54 ± 35.93	597.51 ± 26.15	0.482
AWT (mm)	0.53 ± 0.06	0.54 ± 0.05	0.653
LVPWT (mm)	0.52 ± 0.06	0.53 ± 0.05	0.629
LVEDD (mm)	3.33 ± 0.44	3.07 ± 0.46	0.154
LVEDDI (mm/g)	0.11 ± 0.01	0.10 ± 0.02	0.294
LVESD (mm)	1.82 ± 0.3	1.68 ± 0.23	0.216
LVFS (%)	45.54 ± 3.71	45.04 ± 2.79	0.708
LV Mass (mg)	40.51 ± 13.32	35.98 ± 11.43	0.367
LVMI (mg/g)	1.29 ± 0.31	1.18 ± 0.28	0.322

* Denotes *P* value obtained by chi square test.

*Myh6-Mcm*: Myosin heavy chain 6-MerCreMer; F/M: female/male; HR: heart rate; bpm: beats per minute; AWT: anterior wall thickness; LVPWT: left ventricular posterior wall thickness; LVEDD: left ventricular end diastolic diameter; LVEDDi: LVEDD indexed to body weight; LVESD: left ventricular end systolic diameter; LVFS: left ventricular fractional shortening; LVM: left ventricular mass; LVMI: LVM indexed to body weight.

## Data Availability

RNA-Seq data have been submitted to GEO and available to the public upon release (GSE180972). The list of differentially expressed gene is provided as an Excel sheet in the supplementary material.
