# Supplementary material for: Effects of tamoxifen inducible MerCreMer on gene expression in cardiac myocytes in mice
Source: J Cardiovasc Aging. Author manuscript; Available in PMC 2022 Jan 24. (PMC8785140; doi:10.20517/jca.2021.30)
Supplement: Supplementary Materials [file NIHMS1770300-supplement-Supplementary_Materials.zip › jca-2021-30-SupplementaryMaterials/Supplementary Figure 1.pdf]

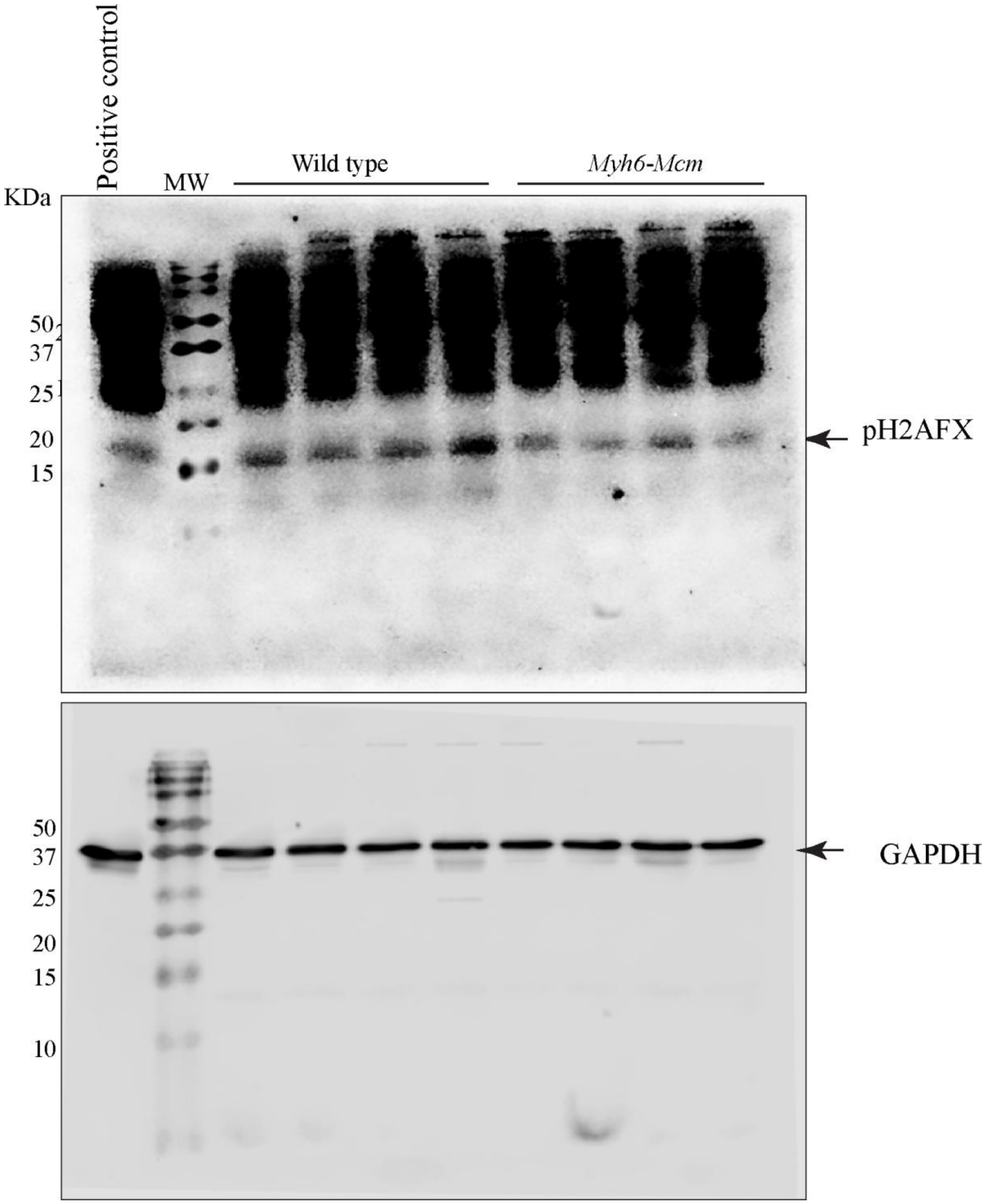

Supplementary Figure 1: The upper gel represents western blot for the detection of pH2AFX and the lower one for the detection of GAPDH, the latter as a loading control.

Molecular weight markers along with a positive control cardiac protein extract from the heart of a mouse with known expression of pH2AFX are included. The upper dark bands are non-specific bands shown upon long exposure of the membrane due to a relatively low expression levels of pH2AFX.
