# Supplementary material for: Effects of tamoxifen inducible MerCreMer on gene expression in cardiac myocytes in mice
Source: J Cardiovasc Aging. Author manuscript; Available in PMC 2022 Jan 24. (PMC8785140; doi:10.20517/jca.2021.30)
Supplement: Supplementary Materials [file NIHMS1770300-supplement-Supplementary_Materials.zip › jca-2021-30-SupplementaryMaterials/Supplementary Table 1.pdf]

Supplementary Table 1: Oligonucleotide Primers, Antibodies, and TaqMan probes

Genotyping primers:

Gene	Forward sequence	Reverse sequence
Cre recombinase	TCTATTGCACACAGCAATCCA	CCAGCATTGTGAGAACAAGG

List of antibodies

Antibodies	Concentration	Supplier	Catalog number
Anti-mouse IgG HRP linked antibody	1:4000 (IB)	Cell Signaling Technology	7076
Anti-Rabbit IgG HRP linked antibody	1:2000 (IB)	Cell Signaling Technology	7074
GAPDH	1:1000 (IB)	Cell Signaling Technology	2118
pH2AFX	1:1000 (IB), 1:200 (IF)	EMD Millipore	05-636

Oligonucleotide primers used in qPCR reactions

Name	Sequence
Gapdh	Forward: AACTTTGGCATTGTGGAAGG<br>Reverse: GGATGCAGGGATGATGTTCT
Lipo2	Forward: ACTCCAACGACTATCAAGCTCC<br>Reverse: TCCACCCATCGATCCCATGA
Ptk2b	Forward:GTGGCCGTCAAGACCTGTAA<br>Reverse: CACGATGTGAGGGTGGTCAA
Abhd1	Forward: GACTTTGCGATAAAGGCCCG<br>Reverse: GGGCTTGCGGCTTGGTAATA
Ptprn2	Forward:GGAACCTGCCCTGGTACATC<br>Reverse: CTGGGGACCCTTGGAACTTG
Mme	Forward: GCTCATTGGTCGGGATGTGT<br>Reverse: CCCATCACCTAAAATCAGTGGGA
If81	Forward: ATTGACCCCAAGCAAGATGT<br>Reverse: ACGGAAGGTGCTCATGTCTG
Gon4I	Forward: CTCAAGACAAGCCACTCGGA<br>Reverse: TGTCTTGCTCTTCGGTTGGG
Arc	Forward: CAGTCTTGGGCAGCATAGCTC<br>Reverse: TGGTATGAATCACTGCTGGGGG
Naip2	Forward: ACAGATCTTCATACTGAAACCACC<br>Reverse: CCTGGGCTGCCATTTTTCTTC
Paqr7	Forward: TCAGCCCATCAGAAGTTTTC<br>Reverse: TGAACTTCTGGGCTACTGCC
A1cf	Forward: ATCACAAATCCGGGGATGGA<br>Reverse: CCTTTGTCCATTTTCCTGGACC

H2-T24	Forward: CCTTGTTCTGAAATGGGAGCCT<br>Reverse: CAGCAAGGTTGCTTGAGGAC
Lrn4	Forward: CAGTATTCCAAAGACTGAGGACC<br>Reverse: CTCTAGGGAAAGACAGGCAGC
Slc46a2	Forward: TGTTCTACATTGCCCGAGCC<br>Reverse: TGGACATGGCTGACCTGATG
Trnp1	Forward: CCTGAAAGTGACACTAAGCTCCT<br>Reverse: GACTGGGTACCGTCAGTCCT
Ler5	Forward: GATCTACAACTCGCGGGTCC<br>Reverse: CACTCAGGTAGACTTGGCGG
Lonrf1	Forward: GAAAACCTCCAGGCAACACC<br>Reverse: CTTCGTCAACCGTTCCTCCA
Per3	Forward: CTCGCCCTACGGTTGCTATC<br>Reverse: CAGTGGACCCTGCTTGAACA
Strit1	Forward: GAAAGACTCCTGGCCCTGAC<br>Reverse: GTGGTGATGTTGACTCTGCTTT
Ctsh	Forward: CCAGGCCTTCGAGTACATCC<br>Reverse: GCAACAGCTTTTTGGGGGTT
Hspb11	Forward: CAGGCGAGCGGGCTAAC<br>Reverse: TCGATGTGGCCAGAATCACTT
Cdsn	Forward: CGGTCTTATTCTGCCAGGAATCT<br>Reverse: TCATTCGGGGAGGTGATCCT
Cpox	Forward: TTCGTGAAGACGTGTGCTGA<br>Reverse: AAACTCCACATACCGCCCTC
Msc	Forward: GGAAAGCATGAAGACGTGGC<br>Reverse: CCATCTAACTGCCCTGTCCC
Mybpc2	Forward: CTGCTAGGGCCTGGTTAGAG<br>Reverse: CCTTTTTGGCCGCTGGTTTA
Mmrn2	Forward: ATTCACAGCAGCCATGTCCA<br>Reverse: TGGGCCACTCGATACATGAC
Mid1ip1	Forward: CGCCCTTACTCACAGTCCG<br>Reverse: TCTAGCCTTTTTCTGCTCTTGC
Adamts19	Forward:GACAATGGTGTCCTGCACAA<br>Reverse: CTGTCATCATCCACCTTGTTTG
Ddit4l	Forward: GAAATCCGGCAGCGCCTA<br>Reverse: TAGCCACCGTCCAACAACTC
Srpr	Forward: GCCATTGCCTTTGCACGTAA<br>Reverse:GCAGTCATCAGAGGGGCATT
Smoc2	Forward: GGAGCAGGGAAAGCAGATGAT<br>Reverse: AACTTGCTCGGTCCAGAGTG
Slc1a4	Forward: TGCCGCATTCACTACGTCTG<br>Reverse: AGGGCAAAAAGGACGAGACC
Lsm11	Forward: GCATCCGAGAGGGAGTCAAG<br>Reverse: CACGTCAGTGAGGGCCATATT
Gpnmb	Forward: AACACAAGGCGTACAAGCCA<br>Reverse: CTGGTCTCCTCGGAAGAACG
Mmp12	Forward: TCTGCTGAAAGGAGTCTGCAC<br>Reverse: AGGTTTCTGCTGGGAACCTTCA
Lgals3	Forward: TACTAGAAGCGGCCGAGC<br>Reverse: TGTCTGCCATTTTCCTGGGTA

Camk2n1	Forward: GGAGCAAGCGCGTTGTTATT<br>Reverse: CAGCCCGCCACTCTTCTTAT
Ctss	Forward: CCACGCTGCCATCAGAAGA<br>Reverse: GATCCCAATGGTAGTCCAGGG
Mpeg1	Forward: AGCCTTCTGACAGAGTCTTGTT<br>Reverse: TCTGCTTCAGCACACGCTAT
Eef1a1	Forward: CGTCGTAATCGGACACGTAGA<br>Reverse: AGGAGCCCTTTCCCATCTCA
Lama4	Forward: CTACACATCAGGGTCGGACG<br>Reverse: CCCATCGTGGTACTGCTTGT
Rpl3	Forward: GGCGGAGCCATCACTTGG<br>Reverse: AGCTGAGAATTTCCTGTGAGACATC
Iqgap1	Forward: CAGCAGTACCAACGACGGTT<br>Reverse: CAGCTGGGCTTCAGGATTCA
Lgals1	Forward: CTTCAATCATGGCCTGTGGTCT<br>Reverse: TGTCTTTTCCCAGGTTCAGCA
Tmsb4x	Forward: GGCCATTTCTAAGTTCCGCC<br>Reverse: AGTCCGGCTTAACTCCTTCG
Ahnak	Forward: TCCCGTAGCTCTGAAGTGGT<br>Reverse: CCACTGCTACAGCCCTGCAT
Atp11a	Forward: GTCTAGCCCAACGCTAGAACA<br>Reverse: GCACACAACTCTCCCAGAGG
Utrn	Forward: AACATCCTCGGCTTGGCTAC<br>Reverse: GGACTGGGAGGGGTCATAGT
Msn	Forward: GGAGTCCTTGCCGAAGTGTA<br>Reverse: ACACGCACACTGATCGTCTT
Clic4	Forward: TGTGCCCACCCAAGTACCTA<br>Reverse: AGCGCTTCATTAGCCTCTGG
Prpf8	Forward: GTTACGGAACCCCACCACAT<br>Reverse: GACGCCACATTCACGTTGTT
Jak1	Forward: TGACCAGGCAAGATCCAGAC<br>Reverse: GACCCTCTCCCAAGTCACGA
Reep5	Forward: TCCTGCACGAGAAGAACTGC<br>Reverse: ACCAGTCCGATGACACCGAG
Mapkapk2	Forward: ATGGGAGGATGTCAAGGAGGA<br>Reverse: CAGAGGGTTGGATGCGTCTT
Canx	Forward: GAGGCCTCTTAGTTCTGCGG<br>Reverse: CCCTTCCATGATCTCGAGCC
Gfm1	Forward: CTCTCCGGATTGCTCGCTTT<br>Reverse: CTCTCCGGATTGCTCGCTTT
Stat3	Forward: GCCCCGTACCTGAAGACCAA<br>Reverse: ACGTGAGCGACTCAAACTGC
Itga9	Forward: GCGTTCTAAGGATTAAGCGGA<br>Reverse: CTTCTTCCAGCGAAGCACTG
Nudt4	Forward: TAGCAGTCGGTACCCAGACC<br>Reverse: CCTTTGACTCCAGCCTCTTCAT
Tfrc	Forward: TAGGCCGCGGGTTCGAG<br>Reverse: GCTACAAGGGAGTACCCCGAC
Arl8a	Forward: GACTGGTTCAAGGCCCTGTT<br>Reverse: TTGAACTGTCCTGACGCGA

C3	Forward: TCCTTCACTATGGGACCAGC<br>Reverse: GGGAGTAATGATGGAATACATGGG
Acta2	Forward: CCAGCCATCTTTCATTGGGATG<br>Reverse: TACCCCCTGACAGGACGTTG
Slc28a2b	Forward: AGCCAGATCTTGTGGCTGG<br>Reverse: TCCTCCAGCATACCGCACT
Ucp3-F1	Forward: ACCCGATACATGAACGCTCC<br>Reverse: AGAAAGGAGGGCACAAATCCTT
Angptl4	Forward: ATTGTTCCAGAAGGTGGCCC<br>Reverse: GCCAAGAGGTCTATCTGGCTC
Pdgfr	Forward: GCTGGAGTCCCTTTCTGGTAAC<br>Reverse: CTGATTCCACGTTGCCATGC
Itih4	Forward: CTGCCCTCCGTTGCACAATA<br>Reverse: CCGGAAGCGGCAAGGTAATA
Alox5	Forward: CGAGTGACAGGGTCAAGAAGT<br>Reverse: GACCGTGTAGGAGGGCATGA
Rorb	Forward: CCTTCTTCCCTCGTGCTGAG<br>Reverse: TCACTTCAATTTGTGCTCGCAT
Ncad	Forward: TCGTGATGGGTGAGGAGACA<br>Reverse: GAGGATCGCCCTGAGAACAC
Tmem82	Forward: TCTGCCAGATACAGGGCTTTC<br>Reverse: TCCATCCTGATTCTCCTTCTGC
Sel1l3	Forward: AGGAGCTCTACGAAAGGTGC<br>Reverse: TGCCGATTGTCTCTCCAACC
Armcx4	Forward: CGAGCAGAGCAGTTCGATGT<br>Reverse: CTCCATAGATCCTGTGCAGC
Atp10d	Forward: ACCTCGGCAAAAGATTGGACT<br>Reverse: CCGGACGGACAATTTCTGGA
Dct	Forward: GTGATCACCACGCAACACTG<br>Reverse: GGGCGTCCTGGACCTAATAA
Gstp2	Forward: AGCACTTGATCCCCACTTCTC<br>Reverse: GGTAACCACCTCCTCCTTCCA
Adam19	Forward: ACAGTTCCTGTCCACACCAAT<br>Reverse: TCTGCTCTGAGTGGATGCTTT
Egf-F1	Forward: GGAGGTCCGCTAGAGAAATGT<br>Reverse: TGGGGCATGTGCAGTGATAG
Adar	Forward: CTTGCCGGCACTATGTCTCA<br>Reverse: CTGCGGGTATCTCCACTTGC
Gbp4	Forward: AGACTTTCCGTGAGGAAGGAT<br>Reverse: TTCCACAAGGGAATCACCATTTT
Ifi27	Forward: GGCCTAGGAGGCAAAAGTCC<br>Reverse: GTTTCCAAACAGGAACCGAATCTTT
Nmi	Forward: GAGATGGACGATATGAGAGGCG<br>Reverse: CGACTGCAATTCAGCTTCAAGT
Trim25	Forward: GTGCGGCCACAATTTCTGC<br>Reverse: GACATTGCGGGCATCGGTA
Parp12	Forward: CATCCTATGGCAAAGGGAGC<br>Reverse: TGACGAAATCTCCGACCAGC
IL18BP	Forward: AGCAGTCCCAACTAAGCAGTA<br>Reverse: CAGCCAGTAGAGGATGCTGA

Ifih1	Forward: GATGTTCTGCGCCAAACTGG<br>Reverse: ACGAGTTAGCCAAGTCTGTGTT
Ier5	Forward: TCACCGCATCGTCAGCATC<br>Reverse: GGGGTCACTCAGGTAGACTTG
Ier3	Forward: CAGCCGAAGGGTGCTCTAC<br>Reverse: AGCCATCAAAATCTGGCAGAAG
Ddit3	Forward: AAGCCTGGTATGAGGATCTGC<br>Reverse: TTCCTGGGGATGAGATATAGGTG
Xpc	Forward: AAAGAAACACCCAAAAAGCAAGG<br>Reverse: CAGCACAGGTTCAGTAAGCTC
Cgrrf1	Forward: GCCGCAGTGTTCCTGGTAA<br>Reverse: TGGTGACGATGAAGCAGGTG

TaqMan probes

**Gene**	**TaqMan Assay ID**
Gapdh	Mm99999915_g1
Myh7	Mm0060555_m1
Nppb	Mm01255770_g1
