# Supplementary material for: Effects of tamoxifen inducible MerCreMer on gene expression in cardiac myocytes in mice
Source: J Cardiovasc Aging. Author manuscript; Available in PMC 2022 Jan 24. (PMC8785140; doi:10.20517/jca.2021.30)
Supplement: Supplementary Materials [file NIHMS1770300-supplement-Supplementary_Materials.zip › jca-2021-30-SupplementaryMaterials/Supplementary Table 2.pdf]

Summary of STAR alignment

Genotypes	Total number of reads	Uniquely mapped reads (N)	Uniquely mapped reads (%)
WT	56,986,661	44,080,962	77.35
WT	58,592,357	45,042,018	76.87
WT	57,741,248	43,997,161	76.2
WT	47,657,324	36,515,508	76.62
WT	51,350,429	36,020,507	70.15
Myh6-Mcm	49,365,176	38,120,087	77.22
Myh6-Mcm	55,698,208	42,618,724	76.52
Myh6-Mcm	52,784,899	37,604,823	71.24
Myh6-Mcm	54,496,135	41,523,760	76.2
Myh6-Mcm	62,083,142	46,430,324	74.79
